# A Potential Integrated Water Quality Strategy for the Mississippi River Basin and the Gulf of Mexico

**DOI:** 10.1100/tsw.2001.354

**Published:** 2001-11-22

**Authors:** Suzie Greenhalgh, Paul Faeth

**Affiliations:** World Resources Institute, Washington, DC, USA

**Keywords:** hypoxia, dead zone, policy, trading, water quality, greenhouse gas, Mississippi River, Gulf of Mexico, climate change, agriculture, environment

## Abstract

Nutrient pollution, now the leading cause of water quality impairment in the U.S., has had significant impact on the nation"s waterways. Excessive nutrient pollution has been linked to habitat loss, fish kills, blooms of toxic algae, and hypoxia (oxygen-depleted water). The hypoxic "dead zone" in the Gulf of Mexico is one of the most striking illustrations of what can happen when too many nutrients from inland watersheds reach coastal areas. Despite programs to improve municipal wastewater treatment facilities, more stringent industrial wastewater requirements, and agricultural programs designed to reduce sediment loads in waterways, water quality and nutrient pollution continues to be a problem, and in many cases has worsened. We undertook a policy analysis to assess how the agricultural community could better reduce its contribution to the dead zone and also to evaluate the synergistic impacts of these policies on other environmental concerns such as climate change. Using a sectorial model of U.S. agriculture, we compared policies including untargeted conservation subsidies, nutrient trading, Conservation Reserve Program extension, agricultural sales of carbon and greenhouse gas credits, and fertilizer reduction. This economic and environmental analysis is watershed-based, primarily focusing on nitrogen in the Mississippi River basin, which allowed us to assess the distribution of nitrogen reduction in streams, environmental co-benefits, and impact on agricultural cash flows within the Mississippi River basin from various options. The model incorporates a number of environmental factors, making it possible to get a more a complete picture of the costs and co-benefits of nutrient reduction. These elements also help to identify the policy options that minimize the costs to farmers and maximize benefits to society.

### INTRODUCTION

The pollution of rivers and estuaries by excessive levels of nutrients such as nitrogen and phosphorus is a persistent water quality problem in the U.S. and a growing problem worldwide. Most of this pollution comes from nonpoint sources, especially agriculture and urban runoff[1]. Some of the most visible impacts of nutrient pollution have occurred in coastal waters and estuaries, where freshwater flows from land meet the ocean. Nutrient influxes in estuaries have increased up to tenfold since the beginning of this century, with the greatest increases occurring after

1950. Scientists have linked these increased nutrient loads with habitat loss, fish kills, blooms of toxic algae, and hypoxia[2].

Hypoxia occurs when the amount of dissolved oxygen in water decreases to levels of 2 ppm or lower. Areas of hypoxia (or "dead zones") are present in more than half of the estuaries of the U.S. One of the largest hypoxic zones off the U.S. coast occurs near the outflows of the Mississippi and Atchafalaya rivers in the northern Gulf of Mexico. This zone, which was 7000 to 10,000 $km^2$ in the summers of 1985 to 1992, doubled to 20,000 $km^2$ in 1999[3], and is now estimated to be larger than 20,720 $km^2$ for 2001[4].

The principal factors leading to the development of hypoxic zones are the stratification of the saltwater/freshwater column and the decomposition of organic matter from nutrient over-enrichment[5,6]. During the summer months, warmer weather and calmer seas cause stratification where the lighter freshwater floats on the seawater, cutting off the flow of oxygen from the surface to the deeper saltwater layer. The nutrient-rich water from the Mississippi River promotes algal growth, which when it dies or is consumed by other aquatic species produces large quantities of organic matter. As the organic matter decomposes it consumes the oxygen in the saltwater layer, thus causing hypoxia. This condition is alleviated in the autumn when stormier weather conditions cause the layers to intermix, allowing oxygen to move through the water column again.

As oxygen stress has increased in the Gulf, the composition of organisms inhabiting bottom waters has shifted over time[7], resulting in fewer fish and a less diverse array of fish inhabiting the area. Fishery managers point out that hypoxia could lead to significant losses for Louisiana, where Gulf fisheries generate more than $2.4 billion of economic activity from recreational and commercial fisheries per year[8]. Despite the current lack of direct evidence of economic impact in the Gulf of Mexico[9], ecological and fisheries impacts of hypoxic zones worsen as they become bigger[10,11] and can cause significant economic impact[12]. The Black Sea, for instance, is now permanently hypoxic below 100 m, and of the 26 commercial fish species only 6 still support a fishery[13].

The productivity and composition of phytoplankton, which are the source of the decaying organic matter, depend on the abundance of nitrogen, phosphorous, and silica in the coastal ecosystem. Of these nutrients, the annual phosphorus flux reaching the Gulf of Mexico is approximately 136,000 metric tons[14]. Although it has not increased significantly over the years, it has large variation among years[15]. Approximately 31% of the total phosphorous flux stems from commercial fertilizers, 18% is from animal manure, and 10% is from point sources. Another 41% comes from sources that have not been quantified, but phosphorus attached to soil particles is believed to be a major component[14].

Silica has a flux of 2.3 million tons, but has no clearly dominant source in the Mississippi River basin[14]. Since 1950, the mean annual concentration of silicates has declined and stabilized[15].

The total annual nitrogen flux from the Mississippi River is approximately 1.5 million metric tons, with nitrates accounting for around 1 million metric tons. This is three times higher than the nitrate flux 30 years ago. Nonpoint sources are thought to contribute as much as 90% of the nitrogen flowing into the Gulf of Mexico, with 56% entering the Mississippi River above the Ohio River. Commercial fertilizer and mineralized soil nitrogen comprise about 50% of the total flux, while atmospheric deposition, soil erosion, and groundwater discharge contribute 24%, animal manure 15%, and point sources 11%. Of these sources, only commercial fertilizer use and legume production have increased significantly since the 1950s[14,16]. As agriculture is the primary source of nitrogen, participation by the agricultural sector in finding a mitigation solution to hypoxia in the Gulf of Mexico is essential in order to achieve the necessary nitrogen loading reductions. Any policy options aimed at reducing the nitrogen flux from the Mississippi River basin will have some economic impact, either positive or negative, on the farming community.

A more detailed discussion of the nutrient composition and the relative ratios of nitrogen, phosphorous, and silica and their importance in the Mississippi River and the continental shelf can be found in Turner and Rabalais[15,17], Rabalais et al.[18], and Rabalais and Turner[6]. It must also be noted that wetland losses, land use changes, and river modification and channelization will also have had some impact of the nutrient delivery to the Gulf of Mexico and are not explicitly considered in this analysis.

### MISSISSIPPI RIVER/GULF OF MEXICO WATERSHED NUTRIENT TASK FORCE

The hypoxic zone in the Gulf of Mexico became a high-priority problem with the establishment in 1997 of the Mississippi River/Gulf of Mexico Watershed Nutrient Task Force, hereafter referred to as the Gulf Hypoxia Task Force. The role of the task force was to study the causes and effects of excess nutrient runoff in the Mississippi River basin and to coordinate and implement nutrient reduction activities to alleviate hypoxia in the Gulf of Mexico. An initial scientific study of the problem resulted in a series of reports by the White House Committee on the Environment and Natural Resources from which an Action Plan was developed. This Action Plan was released in January 2001[19]. To date, the Action Plan has had no funding authorization from the U.S. Congress.

The central coastal goal of the Action Plan was that "by the year 2015, subject to the availability of additional resources, [to] reduce the 5-year running average areal extent of the Gulf of Mexico hypoxic zone to less than 5,000 square kilometers through implementation of specific, practical, and cost-effective voluntary actions by all States, Tribes, and all categories of sources and removals within the Mississippi/Atchafalaya River Basin [and] to reduce the annual discharge of nitrogen into the Gulf[19]."

Model simulations from the scientific reports commissioned by the Gulf Hypoxia Task Force suggest that nutrient (nitrate) load reductions of between 20 to 30% would be sufficient to increase the bottom water dissolved oxygen concentrations by 15 to 50%[20] and meet the Action Plan's coastal goal. Some of the options to reduce nutrient runoff to surface waters include improving the efficiency of farming practices, restoring wetlands, establishing riparian buffers, and tighter controls of point sources such as wastewater treatment plants. Many of the nutrient mitigation options available to reach this target reduction level will also provide local water quality benefits by reducing phosphorus losses.

An economic analysis of the agricultural nutrient loading and hypoxia was commissioned for the Gulf Hypoxia Task Force[21]. This analysis explored a variety of options and their cost-effectiveness using a sectorial model of U.S. agriculture. The version of the model used, while the best available at the time, had a number of deficiencies that limited its utility to address the hypoxia issue. First, the model was not configured by watersheds, making it difficult to draw conclusions about the economic and environmental impacts in the five major sub-basins of the Mississippi River and to assess the nutrient loadings and loading reductions within and from the Mississippi River basin. Second, current industrial and municipal point source information and their level of nutrient removal treatment was not explicitly included in the model. In assessing the feasibility of nutrient trading or tighter regulatory controls, it is important to use up-to-date point-source nutrient discharges and level of nutrient removal treatment. Finally, nitrogen transmission losses to the system as it moves down the Mississippi River to the Gulf of Mexico was not accounted for. The intent of this study was to extend the modeling system to allow it to better address issues relating to the hypoxic zone in the Gulf of Mexico.

### MODELING APPROACH

To evaluate water quality strategies for the Mississippi River basin and the Gulf of Mexico, we used the U.S. Regional Agricultural Sector Model (known as the USMP), a model developed and maintained by the U.S. Department of Agriculture/Economic Research Service (USDA/ERS). This is the model used for the economic analysis commissioned by the Gulf Hypoxia Task Force.

The USMP is designed for general-purpose economic, environmental, and policy analysis of the U.S. agricultural sector. This model is linked to a number of national databases: the regularly updated USDA production practices surveys, the USDA multiyear baseline, and geographic information systems databases such as the National Resources Inventory. The USMP estimates how policy changes, demand, or technology will affect the regional supply of crops and livestock, commodity prices, use of production inputs, net farm returns, government expenditures, participation in farm programs, and environmental indicators. The model maximizes net social benefits in terms of producer and consumer surplus and is constrained by a series of resource and environmental/commodity/input balance constraints.

The World Resources Institute (WRI) has collaborated in the past with USDA/ERS to improve the spatial delineation of the USMP, increase the diversity of cropping rotations, and to simulate the environmental impact of each cropping production practice and the Conservation Reserve Program. The model includes ten major crops (corn, sorghum, oats, barley, wheat, rice, cotton, soybeans, hay, and silage), a number of livestock enterprises (dairy, swine, poultry, and beef cattle), and a variety of different processed and retail products. There are 45 production regions in the model, which are derived from the intersection of the USDA farm production and land resource regions, and approximately 850 different cropping rotations considered for the U.S. based on the various crops and regions.

A majority of the environmental impacts for each cropping rotation are derived using the Erosion/Productivity Impact Calculator (EPIC)[22,23]. EPIC is a crop biophysical simulation model used to estimate the impact of management practices such as fertilizer rate and crop rotations on crop yields, soil quality, and a variety of environmental parameters like nutrient, pesticide, and soil losses at the farm field level. The algorithms for determining these losses from the various cropping activities can be reviewed at http://www.brc.tamus.edu/epic/documentation/index.html. Additional environmental parameters calculated with the USMP model include some greenhouse gas emissions, soil carbon flux, energy use (including that embodied in the production of inputs), and related off-site soil damage.

The dynamics of the nitrogen and carbon cycles are very complicated. For the purpose of this analysis the only sources of cropland nitrogen losses to water considered are nitrate losses from leaching, sediments, surface runoff, and subsurface flow. Sequestered soil carbon is determined from the total organic matter in soil. Nitrous oxide emissions from fertilizer use were derived using the same method as the U.S. Environmental Protection Agency (USEPA) Greenhouse Gas Inventory[24] and calibrating to its estimate.

### MODIFICATIONS TO THE USMP MODEL

Alexander, Smith, and Schwarz[25] showed that the delivery of nitrogen from inland point and nonpoint sources is not a simple function of the distance from these sources to the coast. They demonstrated that the amount of nitrogen delivered from interior watersheds depends on the size of the channels through which nitrogen moves, with the rate of nitrogen loss (or attenuation) in waterways decreasing as channel size increased. This means, in the case of the Mississippi River, that a larger portion of the nitrogen entering the system in the Upper Midwest and traveling through wider streams may reach the Gulf of Mexico than nitrogen traveling through smaller streams close to the Gulf.

Significant sources of nitrogen and phosphorus come from municipal wastewater treatment plants and industrial facilities within the basin. A study initiated by USEPA to determine the total nutrient discharge level from these point sources using 1996 National Pollutant Discharge Elimination System (NPDES) information showed there were about 11,500 permitted facilities in the basin. The discharge rate varied from campgrounds at approximately 0.01 metric tons of nitrogen per year to the Chicago municipal wastewater treatment plant that discharges approximately 10,000 metric tons of nitrogen per year. The estimated total discharge level from point sources in the Mississippi River basin was 286,400 metric tons of nitrogen per year and 59,000 metric tons of phosphorus per year[14].

Watershed delineation, nitrogen attenuation coefficients, and updated point-source discharges are some of the modifications to the USMP version used by the Gulf Hypoxia Task Force economic analysis. The spatial delineation of watersheds within the Mississippi River basin is based on U.S. Geological Survey eight-, four-, and two-digit hydrological units. This enables the economic and environmental parameters to be explicitly determined for the Mississippi River basin. To account for the loss of nitrogen as it moves through the basin, the attenuation coefficients derived using the SPARROW model[25] were included into the model. This information, combined with the watershed delineation, provides more accurate information on the amount of nitrogen reaching the Gulf of Mexico from the Mississippi River sub-basins. In addition, 1996 point-source discharges and their treatment levels determined by the USEPA commissioned study and additional point source facilities identified from the NPDES database or directly from state databases were incorporated into the model[26].

### WATER QUALITY POLICY OPTIONS FOR THE MISSISSIPPI RIVER BASIN

Any successful water quality strategy for the Mississippi River basin must involve participation from the agricultural sector.

A key consideration is finding the most effective way of involving agriculture to achieve reductions in nitrogen flux to the Gulf of Mexico with the least impact on the agricultural community. There are a number of additional environmental co-benefits that can also be gained from strategies aimed at addressing the hypoxic zone in the Gulf of Mexico. Faeth and Greenhalgh[27] showed that strategies aimed at reducing greenhouse gas emissions had significant water quality benefits. Considering these co-benefits as part of the solution set provides a more comprehensive assessment of overall environmental improvements when determining the appropriate strategies to adopt.

A number of scenarios aimed at improving water quality or reducing greenhouse gas emissions were tested to determine their impact on the nutrient load at the mouth of the Mississippi River and on agricultural cash flows.

#### Nitrogen Fertilizer Tax

A significant portion of the nitrogen lost to water in the Mississippi River basin comes from fertilizer. In many instances, farmers apply "insurance" fertilizer rates hoping that climatic conditions produce a bumper crop. In years when growing conditions are not ideal, the crop does not use this "insurance" fertilizer. Frequently, the nitrogen is lost to the atmosphere, leaches into groundwater, or moves with subsurface or surface drainage to waterways.

Tax rates that resulted in a 70 and 500% increase in price were used in this analysis. The 70% tax rate corresponds to the increase in nitrogen fertilizer price observed between 2000 and 2001 due to the limited availability of natural gas (a major component for the production of nitrogen fertilizer) and the corresponding increase in energy prices. The 500% tax corresponds to the tax rate in the Gulf Hypoxia Task Force analysis that achieved fertilizer use reductions resulting in a 20% decrease in nitrogen losses to waterways. This loss value includes nitrogen losses in solution (via surface runoff), nitrogen losses with sediments, nitrogen leaching potential, and nitrogen losses in subsurface flows.

#### Conservation Tillage Subsidies

Tillage subsidy payments have been used for many years to encourage farmers to convert from conventional and moldboard tillage practices to conservation tillage practices. In this analysis, a payment of $25/acre was given for changing to ridge tillage, mulch tillage, or no-till practices. In the past, conservation tillage subsidies were frequently paid on a 75% cost-share basis. Suggested subsidy payments to provide incentives for conservation tillage adoption varies from $10/acre in parts of the Cornbelt and Lake states to $25/acre for cotton acreage in the Southern Plains and Appalachia regions[28]. A payment of $25/acre was chosen for the model since this amount should provide sufficient incentive for farmers to change tillage practices in a majority of regions across the U.S. There was no restriction placed on the type of conservation tillage practices implemented, acreage limits on adoption, or specific areas targeted.

#### Conservation Reserve Program

The Conservation Reserve Program (CRP) was instituted in the 1986 Farm Bill to take marginal, highly erodible land out of production to reduce soil erosion and improve water quality. At the end of 2000, there were 31.4 million acres enrolled in this program. CRP land is not tilled and does not use fertilizer so any increase in CRP would decrease the amount of nitrogen, phosphorus, sediments and pesticides lost to waterways. Greenhouse gas emissions also decrease on CRP land as there is less nitrous oxide emissions from fertilizer applications, no carbon emissions related to tillage operations or the production of fertilizers, and more carbon sequestered in the soil due to the lack of soil disturbance. This analysis allowed CRP acreage to increase to 40 million acres, the acreage cap being proposed by environmental groups for the 2002 Farm Bill, and included an across-the-board increase in rental rates of 20%. The Farm Bill is the piece of legislation that governs agricultural programs in the U.S. and is revised every 6 years.

#### Carbon Trading

Agricultural soils sequester carbon. Tillage practices that cause little soil disturbance, such as no-till, sequester larger amounts of carbon than conventional tillage practice. Different crop rotations also affect the rate of soil carbon sequestration. The trading of soil carbon credits generated by agriculture has the potential to reduce overall U.S. greenhouse gas emissions. In a previous study, Faeth and Greenhalgh[27] showed that strategies to reduce greenhouse gas emissions also provided water quality cobenefits. Many agricultural practices that increase soil carbon sequestration also have significant water quality benefits. For instance, CRP land sequesters large amounts of carbon and lacks the nitrogen loss—both to water and as nitrous oxide to the atmosphere—associated with cropland fertilizer applications. A carbon credit price of $23/t was used to simulate a carbon trading system. This price corresponds to the U.S. Administration's high-end assessment of the carbon permit price if the Kyoto Protocol was implemented[29].

#### Greenhouse Gas Trading

Agriculture is responsible for 11% of the total U.S. emissions of greenhouse gases. Even though carbon dioxide accounts for 80% of U.S. greenhouse gas emissions, agriculture's share of this is only 2%. By far the greatest emissions by agriculture are from nitrous oxide, primarily from fertilizers, and methane from animal-waste handling and rice production. Not only do 74% of nitrous oxide emissions come from agriculture, but nitrous oxide has a heating potential 310 times greater than carbon dioxide. Similarly, methane from agriculture contributes approximately 30% of the total U.S. emissions and is 80 times more powerful than carbon dioxide. Implementing a trading program that addresses all three major greenhouse gases provides greater opportunities for agriculture to reduce its overall emissions. Including nitrous oxide emissions in a trading program also provides direct benefits for reducing the hypoxic zone in the Gulf of Mexico, since a majority of these emissions come from nitrogen fertilizer. As with the carbon trading scenario, a credit price of $23/t was used. It is assumed in this analysis for carbon and greenhouse gas trading that the reduction in carbon dioxide emissions from reduced energy use and the carbon sequestered by soils is permanent. Similarly, nitrous oxide reductions are permanent.

### Nutrient Trading

This market-based mechanism is being explored by a number of state and federal agencies to reduce the cost of improving water quality in such areas as Michigan, the Chesapeake Bay, and Idaho. This concept derives from the fact that each industrial facility or municipal wastewater treatment plant faces different compliance costs depending upon size, scale, age, and overall efficiency. Therefore, the cost of meeting water quality standards may be cheaper for one facility than another. This provides an opportunity for those facilities whose costs are lower to make additional reductions beyond their obligation, and sell these additional reductions to facilities whose costs are higher.

Trading can also occur between a point source such as a municipal wastewater treatment plant and a nonpoint source such as a farm. Point-source facilities are generally controlled by discharge permits mandated by the USEPA, while nonpoint sources are usually not controlled by regulatory limits. As an adjunct to regulation, trading can lower the overall cost of compliance. Web sites such as NutrientNet (http://www.nutrientnet.org/prototype/html/index.html) are being used to reduce the transaction costs of these programs.

The inclusion of nonpoint sources such as agriculture in trading programs has raised the question of uncertainty in the amount of reduction actually achieved by these sources. For agricultural nonpoint sources to reduce their nutrient contribution to water bodies, some kind of best management practice (BMP) would be implemented. These practices may include changing tillage practices or crop rotations, reducing fertilizer rates, or creating filter strips, and can frequently improve water quality at a lower cost than upgrading wastewater treatment facilities. Trading ratios or discount factors are used to account for the uncertainty surrounding nonpoint-source nutrient reductions. A more detailed description of nutrient trading can be found in Greenhalgh and Faeth[30] and Faeth[31].

For this analysis the trading ratio is set at 2:1, meaning that a point source needs to purchase 2 lb of nutrient reduction generated by a nonpoint source for every pound of reduction they require. It is further assumed that the wastewater treatment plants in the Mississippi River basin will be regulated to a 3 mg/l daily discharge of nitrogen. The cost of upgrading wastewater treatment plants to attain the 3-mg/l level is determined from cost curves derived for the Chesapeake Bay by the USEPA[32]. These curves are then used to determine the nitrogen discharge reduction required by wastewater treatment plants in each eight-digit hydrological unit in the Mississippi River basin to met the more stringent standard, the cost of achieving these reductions if the total cost was borne by the wastewater treatment plants, and the amount of nitrogen reduction credits that can be traded in each sub-basin.

## FINDINGS

The findings from this analysis suggest that none of the scenarios alone produced sufficient nitrogen flux reductions (20 to 30%) to the Gulf of Mexico to reduce the size of the hypoxic zone in the Gulf of Mexico to under 5000 km$^2$.

### Nitrogen Fertilizer Tax

A uniform tax on nitrogen fertilizer at the 70% level results in decreases in nitrogen application rates in the Mississippi River basin by about 9%, which relates to a 3% reduction in nitrogen loadings to the Gulf of Mexico. There are corresponding decreases in farm net cash returns and crop acreage under this scenario because of higher input costs. The associated environmental benefits include reductions in greenhouse gas emissions, erosion rates, pesticide losses, and phosphorus runoff to waterways. Using a 500% tax on fertilizer prices resulted in a 40% reduction in nitrogen fertilizer application, with approximately 13% less nitrogen reaching the Gulf of Mexico. The corresponding decreases in farm income and crop acreage and improvements in other environmental co-benefits are of higher magnitude than the 70% tax rate. This scenario produced considerable reductions in greenhouse gas emissions compared to the other policy options because of the large decrease in nitrous oxide emissions from nitrogen fertilizer. The 500% tax on nitrogen fertilizers, however, would not be a feasible policy option for reducing the size of the dead zone because of the substantial decrease in farm net cash returns.

### Conservation Tillage Subsidy

Untargeted conservation tillage subsidies have some environmental benefits but do lead to decreases in farm income. Providing incentives to change tillage practices puts more land into production, which leads to increases in crop production and reduction in crop prices. Nitrogen fertilizer use declines as a result, reducing nitrogen flux at the mouth of the Mississippi River. Greenhouse gas emissions and pesticide losses, however, increase due to increased crop acreage. Erosion decreases, as expected with increases in conservation tillage.

### Conservation Reserve Program

Increasing the rental rate for CRP acreage leads to an increase in CRP acreage and a decrease in crop acres. The increase in overall net cash returns in the Mississippi River basin results from the increase in CRP payments and crop prices. There are small reductions in nitrogen, phosphorus, pesticides, and soil losses to waterways. Larger decreases in greenhouse gas emissions relate to larger amounts of carbon sequestered on the additional land in CRP. The reduction of nitrogen to the Gulf of Mexico is small, around 1%.

### Carbon Trading

The trading of carbon credits and a 70% tax on nitrogen fertilizer result in similar reductions in the nitrogen load reaching the Gulf of Mexico. Reductions in phosphorus and pesticide runoff are greater than the 70% nitrogen tax, conservation tillage subsidies, and CRP expansion would effect, while the decrease in soil erosion is greater than those achieved with the expanded CRP but less than with all the other scenarios tested. As expected, greenhouse gas reductions are greater than most scenarios except for greenhouse gas and nutrient trading. Increases in net farm returns are greater than with the 70% nitrogen fertilizer tax, conservation tillage subsidies, or CRP expansion.

### Greenhouse Gas Trading

The reductions in nitrogen reaching the Gulf of Mexico, phosphorus, pesticide, and soil losses, and the increase in net cash returns are greater than most scenarios except for nutrient trading. As expected the impact that greenhouse gas trading has on greenhouse gas reductions is the best of the scenarios tested. This scenario has the greatest increase in CRP enrollment due to the ability of CRP land to sequester carbon and the lower nitrous oxide emissions resulting from the lack of nitrogen fertilizer applications.

### Nutrient Trading

Permitting wastewater treatment plants to achieve a 3-mg/l discharge level and implementing a nutrient trading program produces the largest reduction in nitrogen flux at the mouth of the Mississippi River, around 12%. Reductions in phosphorus, pesticides, and soil losses to waterways are higher than the other scenarios, while the decreases in greenhouse gas emissions are greater than in all other scenarios except greenhouse gas trading. Net cash returns for farmers also increases more than in most other scenarios. This increase relates to the reduction in crop production from land moving into CRP, the corresponding increases in crop prices that result from decreased supply, and the direct credit payments received for reducing nitrogen lost to waterways. Reductions in nitrogen losses to waterways are easier to achieve with greater enrollment in CRP, since the land enrolled does not receive large nitrogen fertilizer applications.

A uniform nutrient reduction credit price was used for this analysis, corresponding to the lowest credit price for the Mississippi River basin. Adjusting credit prices to correspond to the average credit price derived for each eight-digit hydrological unit may increase the number of nutrient reduction credits traded, achieving an even greater decrease in nonpoint source nitrogen loss to waterways.

Implementing a joint nutrient and greenhouse gas trading program produces similar reductions in nitrogen reaching the Gulf of Mexico as nutrient trading alone. However, this scenario provides greater climate benefits and greater net cash returns for the farmer.

### SUMMARY

No one scenario produced nutrient loadings sufficient to met the Gulf Hypoxia Task Force Action Plan’s coastal goal. To meet this goal, nitrogen (nitrate) loading reductions of 20 to 30% to the Gulf of Mexico are needed. The scenarios tested individually here gave between a 1 and 12% decrease in nitrogen delivered to the Gulf of Mexico. Given that this analysis focuses on nitrate inputs from cropland to the Mississippi River basin, the inclusion of livestock nitrogen losses and wetlands or the introduction of total maximum daily load regulations by the USEPA could mean that reaching the Action Plan’s coastal goal is feasible. Similarly, a combination of policy options could provide greater reductions in nitrogen delivery to the Gulf of Mexico.

By taking into account the attenuation (or loss within the riverine ecosystem) of nitrogen as it moves down the basin, the impacts of improved nutrient management through more efficient nitrogen fertilizer use, and changes in cropping and tillage practices (depending on where they are located in the basin) are diluted. For instance, nitrogen reductions in the Arkansas-White-Red region have higher attenuation rates than does the Upper Mississippi Region[25]. Targeting those sub-basins with higher nitrogen delivery rates and greatest nitrogen contribution to waterways will produce the greatest reduction in the amount of nitrogen reaching the Gulf of Mexico.

The nutrient trading program for nitrogen produces greater reductions in nitrogen fertilizer use in the Upper and Lower Mississippi sub-basins than do the other scenarios. These sub-basins have high nitrogen delivery rates, so decreases in nitrogen fertilizer use will lead to greater reductions in both nitrogen losses to waterways and in nitrogen flux at the mouth of the Mississippi River. In addition, substantial improvements in local water quality from reduced phosphorus, pesticide, and soil loss occur in most sub-basins. Similarly, greenhouse gas emission reductions achieved under nutrient trading scenarios range from 3 to 15% in all Mississippi River sub-basins, highlighting the synergies between water quality improvement and climate change mitigation strategies.

The greenhouse gas and carbon trading scenarios do not produce the same levels of improvement in water quality that are seen with nutrient trading, but the benefits are still greater than in the other scenarios. Climate change improvement, though, is more substantial overall. Most sub-basins except for the Tennessee and Lower Mississippi sub-basins have greater reductions in greenhouse gas emissions under these trading programs than with nutrient trading.

Net cash returns to the agricultural sector tend to decline when a nitrogen fertilizer tax is applied or untargeted conservation tillage subsidies are implemented. The other scenarios induce higher net cash returns, with nutrient and greenhouse gas trading exhibiting the largest increases.

Trading strategies, both for water quality and greenhouse gas reductions, produce greater overall benefits for the environment and for farm returns than do traditional policy approaches. Trading not only exploits the synergistic co-benefits between water quality and climate change but also provides a voluntary incentive mechanism for the agricultural community to be part of the solution to the dead zone in the Gulf of Mexico. Further explorations of combinations of strategies and policy scenarios, with the addition of wetlands and livestock nitrogen losses, may also provide additional policy solutions to effectively meet the Gulf Hypoxia Task Force Action Plan's coastal goal.

### ACKNOWLEDGMENTS

The authors would like to thank Mark Peters and Dina Li from the UDSA Economic Research Service for their assistance with model development and updating the parameters of the model, and Alan Isaac for data collection. We would also like to thank members of the Mississippi River Basin Alliance and the Mississippi Riverwise Partnership for their feedback on the various policy options tested. Lastly, this work would not have been possible without financial assistance from The Oak Foundation, the Curtis and Edith Munson Foundation, The McKnight Foundation, and The Joyce Foundation.

| | | |
|---|---|---|
| Received: | July | 27, 2001 |
| Revised: | October | 9, 2001 |
| Accepted: | November | 1, 2001 |
| Published: | November | 22, 2001 |

### BIOSKETCHES

**Suzie Greenhalgh** is a senior associate in the Economics Program at the World Resources Institute. She holds a Ph.D. in resource economics from The Ohio State University, U.S., a Masters in Economics from The Ohio State University, a Masters in Rural Science from the University of New England, Australia and a Bachelor of Agricultural Science from the University of Queensland, Australia. Her current research interests lie in water quality and climate change issues both in the U.S. and internationally, bio-energy and bio-fuel options for U.S. agriculture and the development of a protocol to assist companies in accounting for greenhouse gas project reductions.

**Paul Faeth** is the Director of the Economics Program at the World Resources Institute. He holds degrees in Agricultural Engineering from the University of Florida and in Resource Policy from Dartmouth College. He currently directs several efforts, including: analysis of the co-benefits of climate policies and water quality; collaborative work with industry on climate change policies; the development of Internet-based trading for nutrients; and an assessment of trade and its impact on the environment in Latin America.
